# Renormalization of metabolic coupling treats age-related degenerative disorders: an oxidative RPE niche fuels the more glycolytic photoreceptors

**DOI:** 10.1038/s41433-021-01726-4

**Published:** 2022-01-01

**Authors:** Nicholas D. Nolan, Salvatore Marco Caruso, Xuan Cui, Stephen H. Tsang

**Affiliations:** 1 Jonas Children’s Vision Care, and Bernard & Shirlee Brown Glaucoma Laboratory, Columbia Stem Cell Initiative, Departments of Ophthalmology, Pathology & Cell Biology, Institute of Human Nutrition, Vagelos College of Physicians and Surgeons, Columbia University, New York, NY USA; 2 Edward S. Harkness Eye Institute, New York-Presbyterian Hospital, New York, NY USA; 3 Department of Pathology & Cell Biology, Institute of Human Nutrition, the Herbert Irving Comprehensive Cancer Center, Columbia University, New York, NY USA

**Keywords:** Medical research, Gene expression

## Abstract

Retinitis pigmentosa is characterized by a dysregulation within the metabolic coupling of the retina, particularly between the glycolytic photoreceptors and the oxidative retina pigment epithelium. This phenomenon of metabolic uncoupling is seen in both aging and retinal degenerative diseases, as well as across a variety of cell types in human biology. Given its crucial role in the health and maintenance of these cell types, the metabolic pathways involved present a suitable area for therapeutic intervention. Herein, this review covers the scope of this delicate metabolic interplay, its dysregulation, how it relates to the retina as well other cell types, and finally concludes with a summary of various strategies aimed at reinstating normal metabolic coupling within the retina, and future directions within the field.

## Introduction

Metabolomics, or the study of fluctuating levels of metabolites used in the production of energy and other life sustaining reactions, is a relatively new tool in medicine and translational research. Its main goal is to identify, quantify, trace, and analyze the levels of these metabolites in real time [1]. This information provides a mechanistic understanding of poorly characterized physiology, and cell–cell interactions that were previously clouded in mystery. Relative metabolite levels compared to normal physiological levels can be a crucial clue to understanding disease progression and underlying pathway involvement. This information is used in both the diagnosis and targeted therapeutic development in many translational research settings today.

One particular metabolic pathway of interest in various cell types is the process of Warburg glycolysis [2]. This process was originally discovered in the 1920s by Otto Warburg, who made observations about large quantities of lactate production in the neuroretinal and tumor cells in the presence of oxygen [2]. This usually occurs as part of an “organized duet” between two compartmentalized cell types. Traditional thinking has taught that one half of this duo, cells rich in glycolytic pathways, results in the end product of pyruvate. Subsequent fate ordinarily depends on the presence of oxygen and mitochondria; however, in certain cell types—including cancer cells, neurons, and photoreceptors—pyruvate is converted to lactate by lactate dehydrogenase. This abundance of lactate generated by these specialized cells feeds other supporting cells that have adapted to use lactate as a fuel source and NAD^+^ to support glycolysis. In addition, Tasdogan et al. showed that lactate uptake mediated oxidative stress in cancerous cells [3].

Until recently, lactate was thought of as mainly a metabolic waste product, but its roles as a carbon source and energy substrate have slowly been uncovered in the past decade. The other half of this coupling phenomenon, oxidative phosphorylation, is responsible for energy production in various cells that act in a supportive manner to the more glycolytic cell types. One particularly interesting coupling phenomena that has been extensively studied is the lactate shuttling that occurs between astrocytes and neurons. In this system, astrocytes sense activity at a neuronal synapse and, as a result, deliver the energy substrate lactate to surrounding neurons [4]. This lactate is used by neurons that generate adenosine triphosphate (ATP) as an energy source, linking the glycolytic astrocytes to the more oxidative neurons in a coupling termed the astrocyte–neuron lactate shuttle. There is also evidence that this behavior is linked to the regulation of blood flow, strongly indicating that astrocytes play a major role in coupling synapse activity to neuronal energy metabolism as a whole. Another notable observation is that the disruption of the metabolic coupling can lead to neurodegenerative conditions such as Alzheimer disease [5] indicating a possible downstream effect in the overall disease progression that is being examined extensively today.

Another occurrence of metabolic coupling observed between two neighboring cell types can be seen in highly oxidative muscle cells, such as those in the heart. Here, lactate is again formed through glycolysis in fast twitch type II muscle fibers. It is then released and consumed by neighboring slow twitch type I muscles, where it is used primarily as a fuel source for the production of ATP in a coupled process that is similar to the astrocyte–neuron shuttle mentioned above [6]. These observations about the role of lactate in physiological metabolic coupling challenge previous notions that lactate is a byproduct of energy production in an oxygen-depleted environment. Instead, they begin to suggest a crucial linking role, also referred to as the “cell–cell” lactate shuttle [7]. This “linking” is fundamental to proper specialized cellular function as described across a variety of cell types, and points to lactate as the connecting metabolite between oxidative and glycolytic metabolism. An example of this lactate coupling is described as follows: lactate is generated in “producer” cells and shuttled to “consumer” cells with a high mitochondrial density and functionality where it serves as a fuel source for ATP/energy production [8]. This cell–cell lactate shuttling is typically seen in muscle cells during exercise and in unique physiological scenarios, such as the previously described astrocyte–neuron lactate shuttle [9]. Yet another example of metabolic coupling occurs during stem cell differentiation in vivo between glycolytic stem cells and oxidative supportive niche cells. Baksh et al. illustrate this coupling in Figure 3 of their manuscript, and show how various nutrient availability could influence ultimate cell fate [10].

### Metabolic coupling in the retina

While there has been considerable interest in gaining understanding in the mechanisms driving metabolic coupling and cell compartmentalization in the brain and across other areas in biology, the retina has piqued the curiosity of many translational scientists. Within the young healthy eye, there is a coupling phenomenon that occurs in the retina, between photoreceptors and the neighboring retinal pigment epithelium (RPE). Photoreceptors undergo a daily renewal process wherein about 10–15% of their outer segments are regenerated to prevent the toxic effects of accumulated byproducts created during phototransduction [11]. This process requires a large amount of both energy and substrate to regenerate the same amount of disk membrane that was shed and digested by adjacent RPE cells. Aerobic glycolysis produces both the energy and substrate necessary to maximize this rate of growth. Therefore, the photoreceptor cells act in a glycolytic manner consuming glucose that is transported by RPE cells from choroidal vasculature and producing significant quantities of lactate through aerobic glycolysis. The photoreceptor cells act in a glycolytic manner, consuming glucose that is supplied by the neighboring choroidal vasculature and producing significant quantities of lactate through a process known as aerobic glycolysis [12]. Before the glucose can reach the photoreceptor cells though, it must first travel through the tightly bound RPE cells, entering through basal transporters and moving down a gradient towards the apical, photoreceptor end [13]. This specialized process supports the rods and cones of the retina’s proper function to transduce light signals into electric impulses to be transmitted and interpreted by the brain. In turn, the mitochondria-dense RPE consumes this lactate amongst other metabolites such as NADH through oxidative phosphorylation, generating vast amounts of ATP. To prevent damage from oxidative stress, the RPE has an abundance of anti-oxidants such as vitamin E, catalase, glutathione (GSH), and ascorbate [14]. GSH acts as a reducing agent by directly quenching oxygen and hydroxyl free radicals as well as peroxides [15]. Other work done by Xiao et al. showed that an increased concentration of lactate in cells limits production of GSH and results in the increase of damaging oxidative species [16]. Conversely, too much reductive stress caused by the excessive accumulation of NADPH or GSH has also shown to have negative effects on cellular function [16]. The abundance of lactate provided by the photoreceptors also serves as a RPE glycolysis suppressor, maintaining the specialized role of the RPE [13].

A schematic illustrating the general metabolic flux in the retina is described by Kanow et al. [13]. This oxidative state allows the RPE to shuttle nutrients to the photoreceptors, protect against photooxidation, re-isomerize trans-retinal, phagocytose photoreceptor-shed segments, and maintain structural integrity of the retina as a whole [17]. Neither of these processes could wholly function in the specialized manner seen in young healthy retina without the other. This metabolic coupling prevents the accumulation of lactate in the retina, and serves to allow the RPE to convert some of this lactate into beta-hydroxybutyrate, which could potentially support the high energetic demands of photoreceptor light transduction [18]. In this light, lactate acts as a linking entity, as previously discussed, between the glycolytic photoreceptors and the oxidative RPE, ensuring proper fuel consumption and a healthy retinal physiology. In addition to supporting the RPE’s proper function, evidence has shown that lactate functions as an energy substrate in Müller cells (MCs) of the retina. Various degenerative conditions such as glaucoma and diabetic retinopathy are typically associated with the death of retinal ganglion cells (RGCs). This cell death is prevented by MCs, which have been shown to require lactate for proper function and health [19]. Evidence has suggested that this lactate consumption is preferential to glucose, and that MCs can even convert lactate to glycogen as a storage mechanism, regulating overall lactate levels in the retina as a whole [20]. This lactate consumption by MCs leads to a positive feedback loop, centered around the expression of the lactate-transporter MCT-1 [20]. In order to keep pace with the high energy consumption demands of this described role, the MCs of the retina have a high mitochondrial density [21]. Many studies have suggested a tightly bound energy production metabolism linking RGCs to MCs, indicating a high degree of energy substrate exchange. RGCs have been shown to consume and secrete lactate, and play a crucial role in preserving visual function and proper retinal metabolism [22].

### Mutations and aging cause loss of metabolic coupling

Researchers have been painstakingly quantifying the metabolite levels in both normal and diseased retinas with the aim of defining “normal” retinal metabolism within the context of photoreceptors, RPE cells, MCs, and RGCs. This is instrumental in understanding the downstream effect of a multitude of gene-specific mutations. Two classes of degenerations in particular, retinitis pigmentosa (RP) and age-related macular degeneration (AMD), have shown many downstream similarities across the range of specific mutations that they are known to be associated with.

**Fig. 1:**
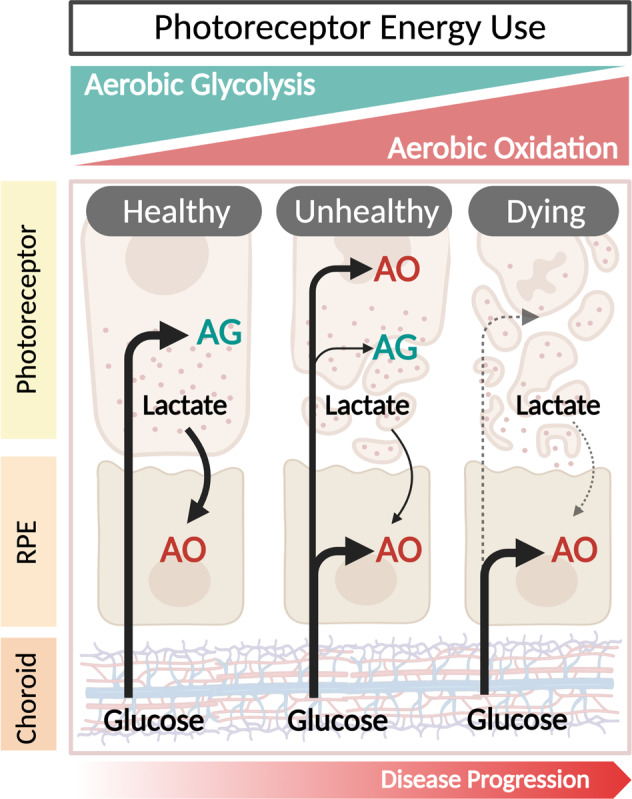
Metabolic coupling of photoreceptors and retinal pigment epithelium (RPE). Pre-disease, the RPE uses lactate from the rods as the preferred carbon source for tricarboxylic acid (TCA) cycle intermediates. Diseased rods shift from aerobic glycolysis to aerobic oxidation, thus decreasing the supply of lactate available to the RPE. In response to this shortage of lactate, the RPE uses the glucose from the choroid that would normally be available to the cones. The cones then atrophy in response to starvation.

RP is caused by any one of over 3100 mutations in 80 genes that, by and large, exhibit rod-specific expression. Any one of these mutations can trigger a progressive pathology of gradual rod loss that corresponds to symptoms of impaired night and peripheral vision. This is followed by the secondary loss of cones, which leads to the more devastating loss of day vision [23–27]. As mentioned above, current knowledge suggests that rods and the RPE are metabolically coupled and that most dystrophic retinas exhibit metabolic uncoupling in both RP and AMD [13, 28–30]. In healthy retinas, rods take up glucose from the RPE and convert it to lactate via aerobic glycolysis. This lactate is then used as a substrate for oxidative phosphorylation and also as a suppressor of glucose consumption in the RPE. In rod dystrophies, on the other hand, the lactate concentration decreases as rod cells die, prompting the RPE to use glucose from the choriocapillaris as an energy source. When nearly all of the available glucose has been consumed by the RPE, cone photoreceptors begin to starve, eventually leading to their death [12]. These findings suggest that photoreceptor death from RP-induced metabolic imbalances could be countered by boosting aerobic glycolysis in dystrophic photoreceptors. Cheng et al. observed that activating the mechanistic target of rapamycin complex 1 in the photoreceptors of wild-type mice, in a manner similar to the metabolic profile seen in AMD-affected retinas, led to early drusen-like formation among other AMD-like pathologies in both the photoreceptors and RPE [31]. An illustrative overview of the major changes to metabolic coupling in the retina, between photoreceptors and the RPE, as a result of many underlying mutations in RP can be seen in Fig. 1. Interestingly, there is considerable overlap in underlying gene mutations between RP and a range of other retinal dystrophies that are also characterized by primary rod and cone photoreceptor degeneration [32]. This seems to suggest similar metabolic pathway dysregulation amongst a host of inherited retinal dystrophies (IRDs). In addition to rod-primary degeneration, there is also mounting evidence that RPE-primary mitochondrial damage leads to a breakdown of the photoreceptor-RPE metabolic coupling [33]. This, in turn, leads to the initial disruption of mitochondrial function and ultimately photoreceptor function. Overall, the major consequence of retinal degenerations is the remodeling of the delicate ecosystem of the retina [34]. This includes the reprogramming of cellular function, rewiring of retinal-neuronal pathways, and altered glial cell response. In addition to in vivo observations, researchers have been diligently working on modeling IRDs in vitro due to the ease of maintenance and ability to study mechanistic pathways involved with these retinal degenerations. Gong et al. found that inducible pluripotent stem cell-derived RPE tissue had a distinct transcriptome from age-matched normal controls, and displayed similar metabolic changes as observed in patient-derived metabolite samples [35].

### Aging mirrors the loss of coupling seen in IRDs

Perhaps the most remarkable finding from studies related to IRDs is that some of the changes observed in late-stage AMD are also observed in the natural aging retina [36]. Wang et al. found that most of the upregulated differentially expressed genes in an aging retina were found in the RPE, endothelial cells, and RGCs. Many of the changes seen in RP and AMD such as decreased *MYC* and mechanistic target of rapamycin (*mTOR)* expression were found in the context of an aging primate retina as well [36]. Single-cell RNA-seq revealed an increase of factors associated with glycolysis in the RPE, and decrease of factors heavily associated with the complementary metabolic state of oxidative phosphorylation. No other major genes were reported as up- or downregulated in the context of aging photoreceptors or RPE. One study looked at oxidative stress in young and aged RPE cells and found an accumulation of mitochondrial DNA damage in the older group from repeated oxidative stress that led to decreased mitochondrial oxidative phosphorylation [37]. Liang et al. found a decrease of the anti-oxidant species mentioned above that correlated with age [14]. Hence, aging causes metabolic uncoupling between RPE and rod photoreceptors, and reinforces the concept that metabolome rejuvenation would provide a therapeutic for age-related disorders.

One solution to restoring the metabolic coupling between the RPE and photoreceptors is to increase hypoxia-inducible factor (HIF) levels. As described by Kierans et al., HIF is responsible for upregulating glucose transporters and glycolytic enzymes [38]. This would subsequently allow mitochondrial DNA damage to shift oxidative phosphorylation to glycolysis in the aging RPE, preventing the consumption of lactate and overall strengthening the argument that the changes observed in the natural aging process mirror the changes that occur in AMD and other IRDs. Yet another study investigated the role of *mTOR*, whose aberrant glycolytic activity is often linked to the natural aging process. Findings from this work concluded that hyperactive *mTOR*-associated signaling led to a progressive RPE degeneration, in line with observations made in the context of retinal degenerations such as AMD [39]. While increased *mTOR* activity was linked to a higher rate of lipogenesis and drusen formation, it came at the cost of an increased glycolysis, which depletes the retinal supply of glucose and ultimately starves the photoreceptors.

### Therapeutic restoration of metabolic coupling

Over the past decade, there has been an enormous effort to develop therapeutic editing tools such as clustered regularly interspaced short palindromic repeats (CRISPR), transcription activator-like effector nucleases, and zinc-finger nucleases to precisely edit the mammalian genome. While all of these methods have been explored, CRISPR has gained the most traction due to its flexibility, versatility, user-friendly platform, and editing efficiency capabilities. The only major limitation of the CRISPR editing system is the requirement of a specific protospacer-adjacent motif near the desired editing site, and because of this a variety of edits are possible and likely to succeed in some form or fashion. While CRISPR-based gene-editing has provided a platform to develop endless gene-specific therapies targeting each of the identified mutations associated with retinal degenerations, also known as precision medicine, the highly diverse pool of underlying mutations (>3100) in RP and risk alleles in AMD compromise the practicality and feasibility of such an approach. Each gene-editing system needs to be custom designed, fine-tuned, rigorously tested, and gain FDA approval, a process that is both time and financially costly. Consequently, there has been tremendous interest in the development of a therapeutic approach that takes advantage of a convergent downstream pathway in many of these underlying mutations [40, 41]. Metabolomics has uncovered many of these therapeutic targets, and also allowed researchers to gain a precise understanding of how the metabolic coupling of various cell types has been altered throughout disease progression and normal aging. Building on this knowledge, various strategies have been explored in recent years, mainly focusing on the lactate-associated glycolysis and oxidative phosphorylation, and *mTOR* -associated pathways as described by Caruso et al. [42]. The authors describe the estimated years of mainly cone vision preservation based on a mouse-to-human years conversion established by Dutta et al. [43].

**Fig. 2:**
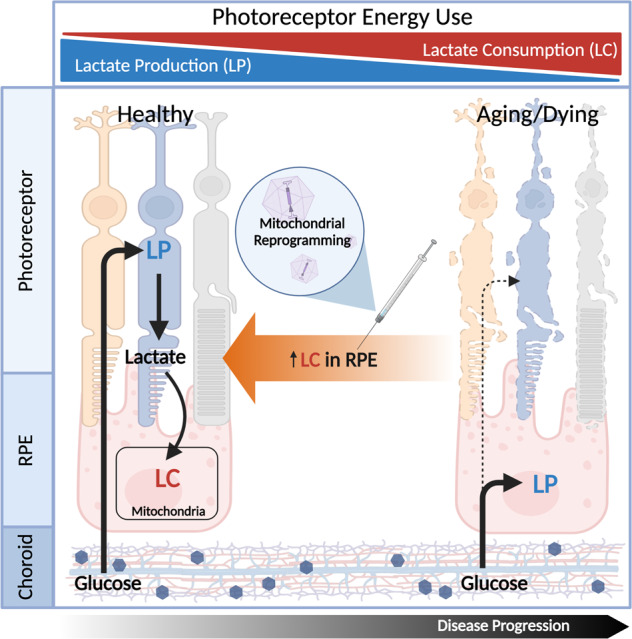
Overall hypothesis. Our hypothesis states that increasing lactate intake and consumption in retinal pigment epithelium (RPE) mitochondria consequently promote photoreceptor survival and reinstate the proper metabolic state in both the RPE and photoreceptors.

Many of the therapeutic targets described by Caruso et al. have shown great promise in enriching a desired metabolic state in a specific cell types, effectively renormalizing the metabolome of the neural retina (Fig. 2) [42]. Interestingly, many of the targeted mutations occur specifically in rods, and lead to a secondary loss of cones [44]. To explore the causal relationship between photoreceptor death in retinal degenerations, scientists investigated changes in gene expression following the onset of these rod-specific mutations. Their research revealed that nearly one third of expression-altered genes played a role in the metabolic *mTOR* pathway [27]. This pathway has been found to be involved extensively in protein synthesis amongst other anabolic processes. Other findings from this work include the observation that cones appear to be starved following the rod-specific mutations, indicating that a metabolic pathway is being disrupted. Inspired by this work, Zhang et al. explored a different strategy aimed at delaying photoreceptor degeneration, focusing on the *mTOR* downregulator tuberous sclerosis complex 1 in a RP mouse model [45]. Findings from this study include the phenotypic and functional rescue of rods and cones in early-stage RP, supporting the idea that cone starvation is preventable following therapeutic intervention. Another study from the same group investigated the role of sirtuin 6 (*Sirt6*), a glycolysis suppressor in the context of an RP mouse model [46]. Rod-specific ablation put photoreceptors in a forced state of glycolysis and preserved outer segment health as well as photoreceptor functionality [46]. Another key regulator of cellular metabolism is pyruvate kinase M2 (PKM2) that downregulates the associated pentose phosphate pathway. Zhang et al. investigated this avenue, ablating *PKM2* and found long-term photoreceptor preservation and an improved retinal functionality compared to untreated RP-background control mice [47]. Another key substrate provided by the rod photoreceptors for proper cone health and function is rod-derived cone viability factor (RdCVF). Byrne et al. explored various forms of viral mediated RdCVF-expression in an RP mouse model and observed improved cone function, delayed cone death, increased rhodopsin mRNA levels, and decreased oxidative byproducts [48]. These findings suggest another possible imprecision medicine therapeutic avenue for treating RP patients from a wide variety of underlying mutations.

A different strategy for renormalizing the metabolic coupling of the retina revolves around microglia, and the inflammatory effect they convey. Transforming growth factor beta (TGF-β) is partially responsible for downregulating this inflammatory response, which was postulated to worsen the cone degeneration observed in RP. By over-expressing TGF-β through AAV-based delivery, Wang et al. were able to downregulate this inflammatory response and ultimately delay photoreceptor degeneration in three separate RP-background mouse models [49]. All of the studies described show great promise as a way to imprecisely target downstream effects of various underlying mutations in retinal degenerations such as RP. One major concern with the metabolic reprogramming deal with safety of the proposed therapeutics. Data have suggested the modulation of photoreceptor metabolism in control mice to have somewhat detrimental effects [46]. However, in the context of RP-associated mutations, there is no evidence of any harmful effects when approaching these suggested therapeutic avenues. There is also no evidence of hemangioblastoma-like lesions in RP-background patients in any of the proposed therapies [50]. Overall, the data suggest that metabolic reprogramming, for the purpose of slowing down/halting retinal degeneration, is safe in the context of an underlying RP-associated mutation.

## Conclusion

Bidirectional metabolic crosstalk between rods and the RPE underscores the need to directly interrogate the metabolic behavior of distinct cell types in live animal models. Developments in metabolomics have uncovered a metabolic coupling between photoreceptors and supportive RPE cells that is essential to the long-term health and function of the retina as a whole. In this review, we explored metabolic coupling between various cell types in the retina, how retinal degenerations progress through the breakdown of this metabolic coupling, how aging mirrors the loss of coupling seen in the degenerative conditions, and lastly the development of strategies aimed at renormalizing the metabolic coupling between photoreceptors and RPE cells as an imprecision medicine therapeutic avenue. Metabolome engineering will serve as an instrumental rejuvenation tool in age-related degenerative conditions including RP and AMD in the years to come, and will likely see use in many fields beyond ophthalmology.
