# Correction: Renormalization of metabolic coupling treats age-related degenerative disorders: an oxidative RPE niche fuels the more glycolytic photoreceptors

**DOI:** 10.1038/s41433-022-02129-9

**Published:** 2022-08-10

**Authors:** Nicholas D. Nolan, Salvatore Marco Caruso, Xuan Cui, Stephen H. Tsang

**Affiliations:** 1 Jonas Children’s Vision Care, and Bernard & Shirlee Brown Glaucoma Laboratory, Columbia Stem Cell Initiative, Departments of Ophthalmology, Pathology & Cell Biology, Institute of Human Nutrition, Vagelos College of Physicians and Surgeons, Columbia University, New York, NY USA; 2 Edward S. Harkness Eye Institute, New York-Presbyterian Hospital, New York, NY USA; 3 Department of Pathology & Cell Biology, Institute of Human Nutrition, the Herbert Irving Comprehensive Cancer Center, Columbia University, New York, NY USA

**Keywords:** Medical research, Gene expression

Correction to: *Eye* 10.1038/s41433-021-01726-4, published online 01 January 2022

The author Salvatore M. Caruso1,2, is listed at pubmed only under his full middle name, so that the name is given in full as Salvatore Marco Caruso, now.

The original article has been corrected.

